# Clog-free high-throughput microfluidic cell isolation with multifunctional microposts

**DOI:** 10.1038/s41598-021-94123-6

**Published:** 2021-08-17

**Authors:** Dilip Venugopal, Nanda Kasani, Yariswamy Manjunath, Guangfu Li, Jussuf T. Kaifi, Jae W. Kwon

**Affiliations:** 1 Department of Electrical Engineering and Computer Sciences, University of Missouri, Columbia, MO 65211 USA; 2 Department of Surgery, Ellis Fischel Cancer Center, University of Missouri, Columbia, MO 65212 USA

**Keywords:** Isolation, separation and purification, Lab-on-a-chip

## Abstract

Microfluidics have been applied to filtration of rare tumor cells from the blood as liquid biopsies. Processing is highly limited by low flow rates and device clogging due to a single function of fluidic paths. A novel method using multifunctional hybrid functional microposts was developed. A swift by-passing route for non-tumor cells was integrated to prevent very common clogging problems. Performance was characterized using microbeads (10 µm) and human cancer cells that were spiked in human blood. Design-I showed a capture efficiency of 96% for microbeads and 87% for cancer cells at 1 ml/min flow rate. An improved Design-II presented a higher capture efficiency of 100% for microbeads and 96% for cancer cells. Our method of utilizing various microfluidic functions of separation, bypass and capture has successfully guaranteed highly efficient separation of rare cells from biological fluids.

## Introduction

Recently, microfluidics-based platforms have been widely integrated into numerous research areas. Examples include microreactors for chemical synthesis^1^, the use of microfluidic technologies for proteomic analysis in samples with low protein content^2^, and advanced cellular research for biomedical and healthcare applications. Significant research accomplishments have advanced biomedical microfluidic platforms to diagnostic and prognostic point-of-care levels^3^. An unmet need in particle-based biomedical research is efficient capture and retrieval of viable cells for downstream liquid biopsy investigations (e.g., drug sensitivity testing of rare circulating tumor cells (CTCs) from cancer patients’ blood), while maintaining a high flow rate and clog-free device features^4^.

Label-based capture relying on biomarker expression is commonly performed for isolation of rare CTCs from an abundance of other blood cells^5^. However, these label-based enrichment methods are limited by a lack of specificity due to inconsistent and heterogeneous cellular biomarker expressions in tumor cells^6^. To overcome these challenges, there have been vigorous efforts to develop microfluidic platforms for label-free separation of cells utilizing intrinsic physical properties (e.g., size, mass, electrical charge)^6,7^. Many microfluidic techniques that incorporate size-based enrichment (such as membrane microfiltration^7^, pinched flow fractionation^8^, deterministic lateral displacement^9^, and hydrophoresis^10^) have the potential for a wide range of applications in biomedicine. Another major challenge is to efficiently capture rare cells in a viable state from various media like aerosols, blood, or other body fluids. For these cell isolations, fluidic sorting operations have been proposed to achieve higher selectivity, specificity, sensitivity, reliability, and quick processing times. But all these important criteria have not been satisfied by a certain method so far.

Based on the principles of actuation and control mechanism, separation methods can be broadly classified into active and passive. For active methods external forces are used to achieve high-resolution particle separation, consistently requiring a particle’s unique physical property. Active methods mainly include electrophoresis^11^, magnetophoresis^12–14^, negative magnetophoresis^13,14^, optical^15^ and thermal manipulations^16^. They typically require high-power energy consumption to generate sufficient external forces for separation based on intrinsic properties^11,17^. In contrast, passive separations depend on the characteristics of the flow in the microfluidic channel. Major advantages are not only the ease of fabrication of the platform but in particular, their independence from external forces and low-power energy consumption for processing. Existing passive separation methods include hydrodynamic filtration^18^, inertial lift^19,20^, deterministic lateral displacement^9^, Dean’s flow^21^, and pinched flow fractionalization^8,20^.

Most of the studies conducted using microfluidic devices have revealed the need for very low flow rates (< 0.05 ml/min) to maintain an acceptable level of capture efficiency, however, these lead to prolonged and unimplementable processing times^22^. There is also a lack of systematic research directed towards imposing various shapes and arrangement of the micropost structures that could innovatively solve problems. Attempts were made by adopting cylindrical^23^ and equilateral triangular microposts^23,24^ to enhance particle capturing. However, during the filtering process, all the microposts in the first row of the array get engaged in particle capturing, leading to serious clogging that eventually arrests the flow and damages the sample.

**Figure 1:**
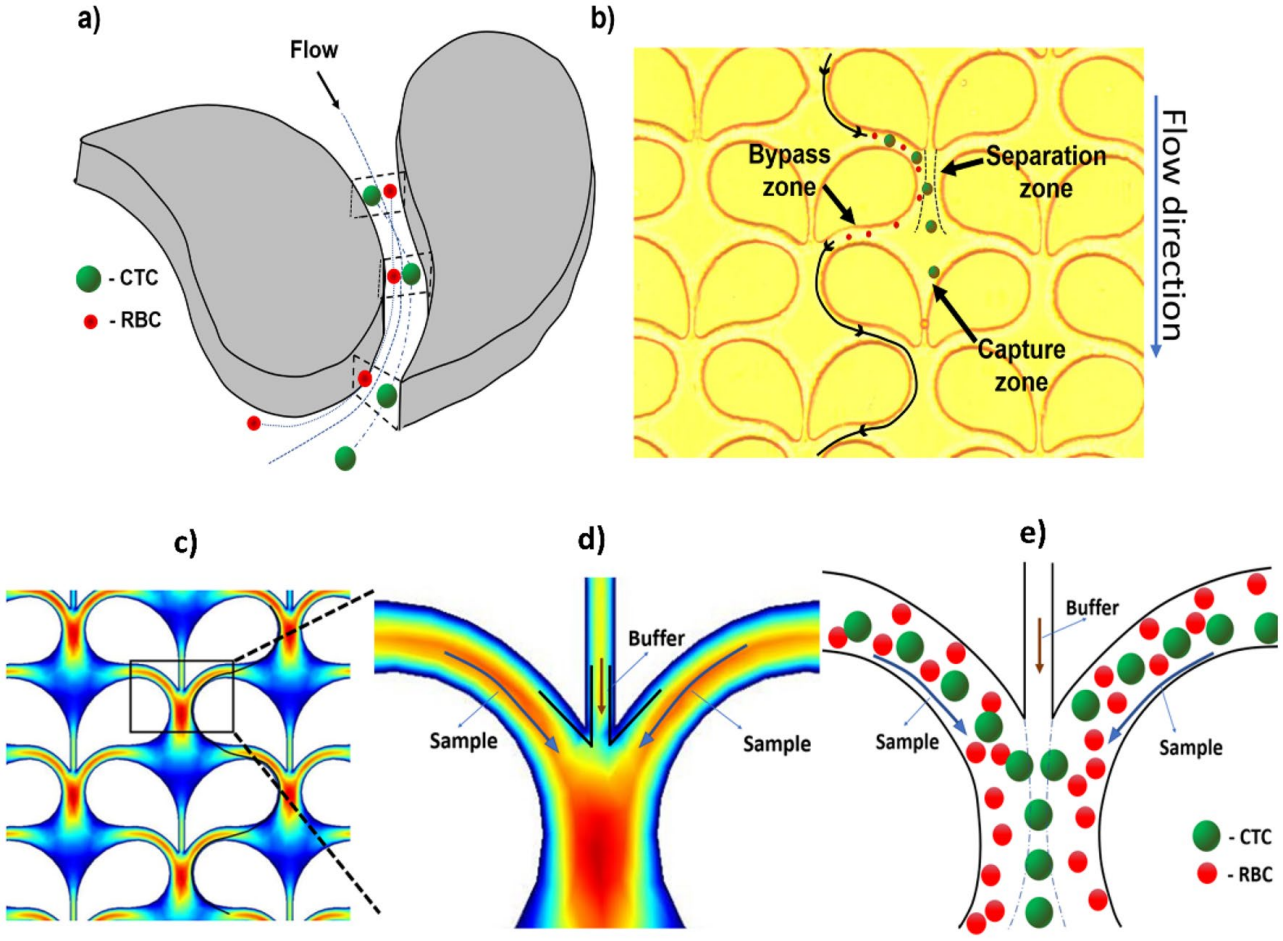
Particle movement within the microfluidic device (Design-I). (**a**) Particle movement around a micropost showing the motion of particles based on their size through the microfluidic channel (CTC: circulating tumor cell (larger particle in green); RBC: red blood cell (smaller particle in red)). (**b**) Top view microscopic image showing the array design with paired and a trajectory of smaller particles through the 14 µm by-passing zone and 7 µm particle capture zone. (**c**) Velocity profile computational simulation (COMSOL software) of Design-I indicating high velocity (red) around the curvature and at the point where they collide. Low velocity (dark blue) is noted around the 7 µm capture site for the particle to safely rest once isolated. (**d**) Magnified view of the focused window of (**c**) showing the velocity profile at the separation zone. The sample is at high velocity due to presence of denser particles (sample flowing around the curvature), and buffer through the capture site channel has lower velocity. (**e**) A particle movement scheme in the microfluidic channels based on particle sizes (CTC: large; RBC: small) in the magnified rectangular region of (**c**).

In this article, we introduce an innovative microfluidic platform with an array of uniquely designed multifunctional microposts to achieve very high capture efficiency and flow rates with the absence of detrimental clogging problems. Especially, an alternative carry-forward path is employed, which allows particles to simply bypass a congested area. Such a bypassing zone (Fig. 1b) seems very effective to release undesired impacts of surge pressure build-up by clogging and to avoid the generation of shear stress that negatively impacts cell viability^25^. By adjusting geometric effects, preventative measures on clogging and efficient capturing of cells were favourably achieved. By applying various approaches, we were able to successfully demonstrate an innovative high-throughput microfluidic isolation method for rare cells from the blood.

## Results and discussion

Our microfluidic isolation method was successfully enabled by the innovative form of microposts with multiple key functions. Each micropost was designed with precisely but unevenly curved shapes and placed in distinct orientations with the unique wing-like arrangement in pairs. Bypassing, separating, and capturing functions were effectively achieved in different parts of the micropost. Each part collaboratively contributed towards ensuring high capture efficiency while overcoming the generic problems of low flow rates and clogging in existing microfluidic technologies. Under various operating conditions, the performance was evaluated for other critical factors, such as repeatability, reliability, and different flow rate with microbeads and human cancer cells.

The separation zone utilizes the inertial migration of microparticles flowing through a curved microchannel. According to W. R. Dean^21,25–28^, in a curved microchannel^21,28^ the fluidic flow around a curvature forms helical streamlines^21,27,28^. These helical streamlines—known as the secondary flow path—are moving away from the curved micropost walls and are deviating from the main flow (Fig. 1a). The secondary flow is relatively miniscule compared to the main flow. The secondary flow depends on both the radius of the curved path and the size of the particle, and therefore the secondary flow can be utilized to separate and capture larger particles by guiding them to the isolation region within the capture site^21,26,27^ (Fig. 1b). By modifying these parameters, we designed a micropost with a sufficiently large Dean number (D_e_) to achieve a high secondary flow to aid the particle movement around the micropost. Figure 1b and Supplementary Information S2 outlines the device with the micropost arrangement within the device with the capture site that has a 7 µm gap, and an additional 14 µm by-passing channel. This distinct design results in an asymmetrical flow pattern. The movement of particles around these high-aspect ratios (h/w) of the microfluidic channel can be explained by the rotation-induced lift force (F_Ω_) which is a resulting factor of secondary flow^29–31^. Firstly, the particles experience shear-induced lift force^29,30^ (F_S_) and move towards the sidewalls of the microposts. Then, due to rotation-induced lift force (F_R_), the particles are pushed away from the walls and an equilibrium is achieved along the long-curved surfaces of the micropost. Due to our unique wing-like micropost design the rotation-induced lift force (F_R_) is predominant, with larger particles experiencing more force due to their density that directs them away from the walls of the micropost towards the capture site^28,32^. For a particle to be considered sufficiently large to experience this force, it must have a diameter greater than 72% of the channel depth^30^. In addition, any smaller particles with diameters less than 27% of the channel depth will not experience enough force to be directed towards the capture site^30^ as indicated in Fig. 1e.

**Figure 2:**
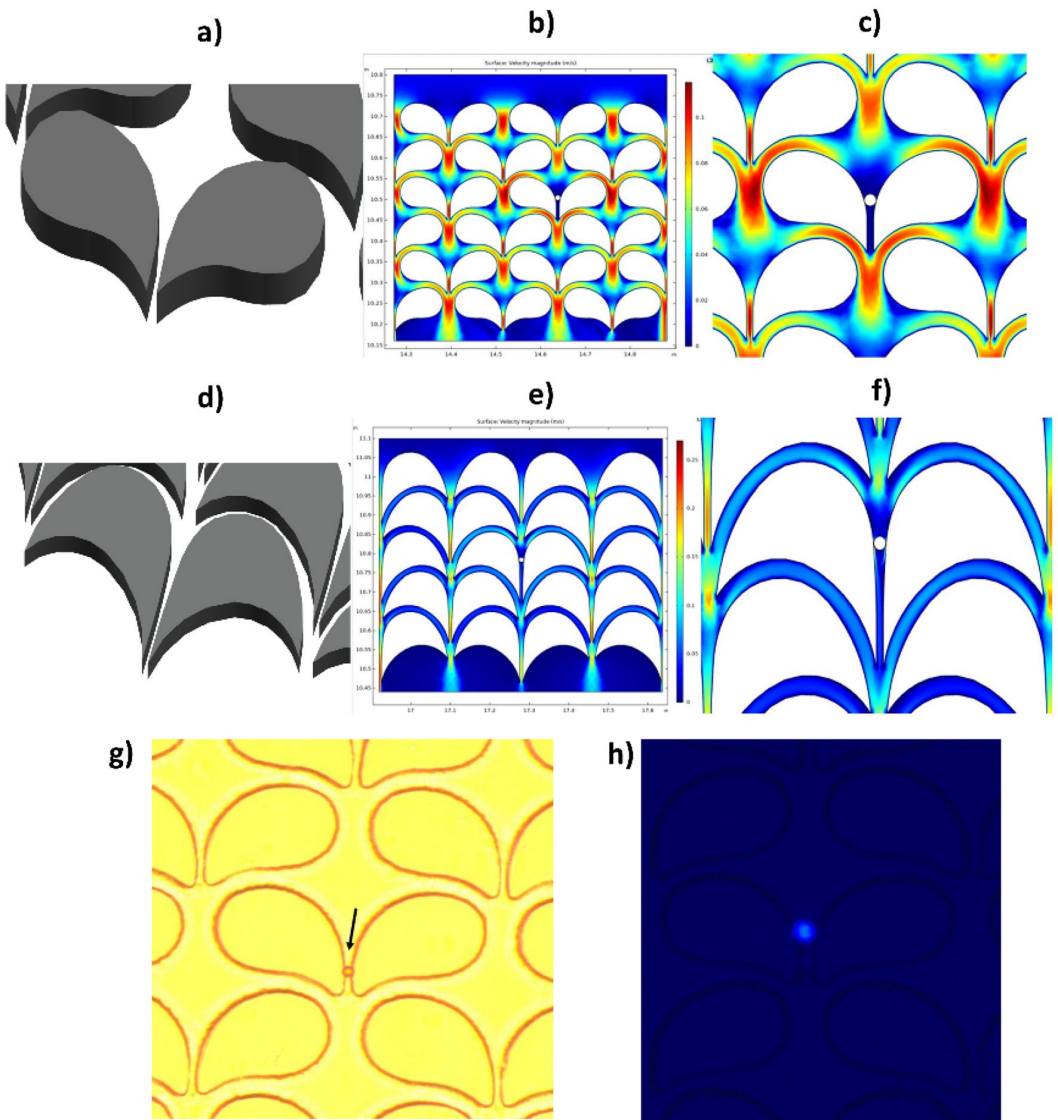
Micropost array designs and velocity profiles. (**a**) Schematic diagram of the micropost array (Design-I) in the device. (**b**) Computational analysis of velocity profile at multiple micropost pairs with wing-like arrangement. The capture site was computationally tested at a maximum velocity (V_max_) which is the highest flow observed in the separation zone of 0.1 m/s. (**c**) Magnified portion depicting velocity profile with a particle caught in the capture site. Particles move radially outward during the movement passage through the curved channel (red arc). (**d**) The schematic diagram of the modified microposts (Design-II) in the curved microchannel. Modified microposts improve the cell trapping efficiency of the capture site. (**e**) Velocity profile by computational analysis of multiple wing-like shaped microposts in pairs. The capture site was computationally tested at V_max_ of 0.1 m/s. (**f**) Magnified image showing the velocity profile with a particle trapped in the capture site. (**g**) Microscopic view of the microbead captured in the device under brightfield. (**h**) Fluorescence microscopic view of a single DAPI-stained cancer cell captured in the device.

The bypass zone and the capture zone are separately located, creating an uneven flow pattern on both sides of the non-symmetric micropost. The capture zone as indicated in Fig. 1b, consists of 7 µm gap where the cell is trapped after being directed by the separation zone. The bypass zone provided two main advantages, one is to transport smaller particles (such as RBCs) and the second for single cell isolation. The former is achieved since smaller particles did not experience sufficient force to be directed towards the capture zone while the latter, when a cell is trapped at the capture zone resulting in no flow through the 7 µm zone and around that region (Fig. 2c,f) helping in redirecting (Supplementary Information S3) new larger particles through the bypass route towards the next available empty capture zone thereby eliminating clogging problem as seen in other microfluidic technologies. Finite element analysis was performed to identify the optimal operating conditions and design for the microposts with wing-like geometry using COMSOL software (Fig. 1c). The operating conditions for the fluidic medium used were similar to that of water, consisting of incompressible flow, inlet linear velocity of 12 mm/s, no-slip boundary conditions for all walls, and an outlet pressure of zero. The representation of the velocity profile in the separation zone of the two by-passing channels as is shown in the thermal graph (Fig. 1d). High velocity was observed above the capture site, serving favorably the purpose to guide the laterally displaced particle towards the capture site (Fig. 1d). Using these parameters both variations of the microposts designs were simulated (Fig. 2b,e) to find the fluidic pattern around the microposts and at the capture zone with a trapped particle.

### Testing performed using microbeads in PBS fluid medium

**Figure 3:**
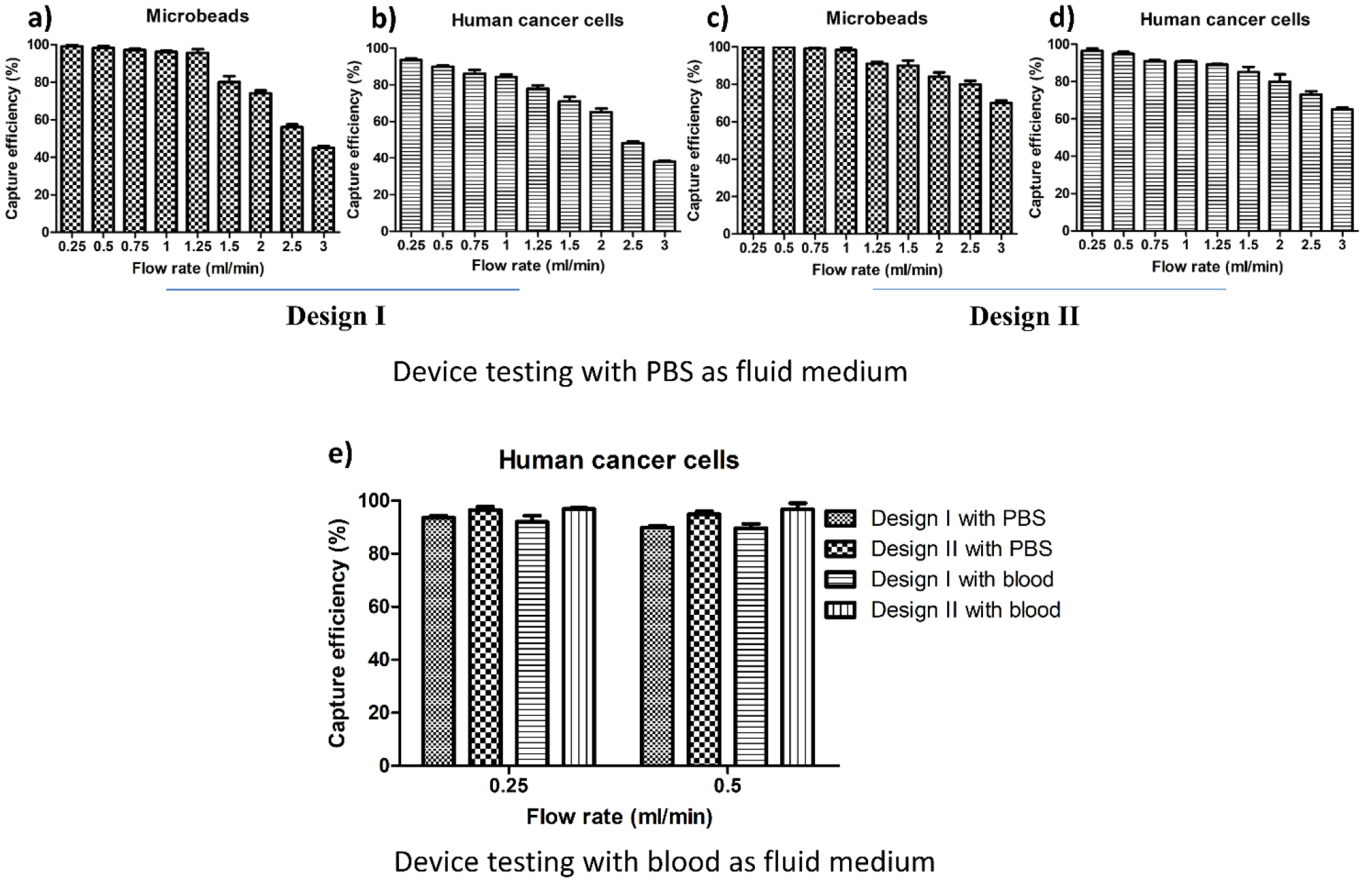
Capture efficiencies for Design-I and Design-II. Capture efficiency as a function of flow rates for (**a**) 100 microbeads spiked in 4 ml phosphate-buffered saline (PBS) for Design-I. (**b**) 100 human cancer cells spiked in 4 ml of PBS for Design-I. (**c**) 100 microbeads in Design-II. (**d**) 100 human cancer cells in Design-II. (**e**) Capture efficiency as a function of flow rates for both Design-I and Design-II tested with 100 cancer cells spiked separately in 4 ml of PBS and diluted human blood (1:5 in PBS). Data bars represent five replicates at each flow rate and each category (error bar: standard error of the mean).

Our prototype microfluidic device (Design-I) and the improved version (Design-II) were tested for capture efficiency at different flow rates using microbeads. Microbeads (Fig. 3a,c) as uniform-sized particles not perturbed by shear stress are routinely used for standardized evaluation of the microfluidic isolation method. Using microbeads in Design-I, a maximum capture efficiency of 100% was observed at a flow rate up to 0.5 ml/min that is already very high to be suitable for biomedical and clinical applications (Fig. 3a). We observed that the capture efficiency dropped significantly at or above a flow rate of 0.75 ml/min. Importantly, there was no clogging observed within the device for all testing performed. Despite observing high throughput parameters with Design-I, an attempt was made to further improve the capture efficiency of Design-I by modifying the shape, geometry, and arrangement of microposts. This improved Design-II exhibited an enhanced performance by achieving a 100% capture efficiency at a flow rate of 1 ml/min with microbeads (Fig. 3c). Of note, the integrity of the device and microposts was also maintained firmly in testing with higher flow rates, indicating stability under different operational conditions.

### Testing performed using human cancer cells in PBS fluid medium

In contrast to fixed-sized microbeads that are stable, cells have different and changing sizes, shape, dynamics, densities, and exhibit plasticity under physiological conditions. These biological properties make cells susceptible to damage and loss of integrity by shear stress due to fluid movement within the device. Design-I was effective in capturing human cancer cells (87% at 1 ml/min) (Fig. 3b). Sufficiently high capture efficiency was maintained at a flow rate of 0.5 ml/min which is significantly higher compared to existing microfluidic methods^22^. Despite their susceptible nature, captured cancer cells within the device appeared microscopically to maintain an intact shape (Supplementary Information S1) which is indicative of their integrity, such as their outer membranes, cytoplasm, and nuclei while moving through the device. Design-II was performed with an improved capture efficiency in accordance with the theoretical aspect of the geometrical design of 96% at a flow rate of up to 1 ml/min with human cancer cells (Fig. 3d). Results clearly depict that Design-II has an improved microfluidic profile in comparison to Design-I because of the detailed modifications that were applied to the geometric shape of the microposts. Of note, the modifications in the microposts for Design-II still retained high-throughput and clog-free features, while maintaining microstructural device integrity and microscopic cancer cell viability.

### Capture efficiency of both designs in the pre-clinical setting

Both designs performed well in capturing human cancer cells spiked in the buffer solution. But to solidify the foundation for our method’s potential applications in real biomedical and clinical settings, we tested both Design-I and -II with human blood as the ultimate fluid medium. Healthy human blood that has a viscosity of 3.5 × 10^−3^ to 5.5 × 10^−3^ Pa s^33^ was diluted in a ratio of 1:5^34^ (blood:PBS) to achieve feasible and applicable fluidic conditions in the device. Although most of the RBCs pass through the device, a negligible fraction of RBCs was retained within the device due to their high numbers (4.1–5.1 million/mm^3^). As expected, the small fraction of RBCs retained in the device did not alter the fluidic flow or cause difficulty in the microscopic enumeration of captured cancer cells. To distinguish human cancer cells from the abundance of white blood cells (WBCs), cancer cells were fixed, permeabilized and nuclear staining was performed with 4′,6′-diamidino-2-phenylindole (DAPI) prior to spiking which allows the identification using fluorescence microscopy (Fig. 2h). Figure 3e shows the efficiency of both designs with either PBS or diluted blood and with each data bar in the graphs representing five replicate tests. Although there was more variability as reflected by the range of the standard error of the means, experimental results obtained with human blood were very comparable to the data obtained with PBS buffer tests. This indicates the potential applicability of the novel microfluidic device design for clinical and biomedical applications involving human blood.

### Leukocyte filtration through the device

Table 1 lists all types of leukocytes and their respective nucleus to the cytoplasmic ratio^35–37^ which indicates that the nucleus size is less than 7 µm so that they can squeeze through the trapping region with little or no effort. A couple of tests were conducted to see how many leukocytes gets trapped in the device compared to an approximated assumption of its presence in human blood (1:5 dilution) infused through the device. The number of WBCs in healthy blood ranges from 4.5 × 10^6^ to 11 × 10^6^/mL. However, we observed that only a negligible fraction (~ 1/10,000 WBCs) of fluorescently-labeled (CytoTracker™ Green) WBCs spiked into healthy whole blood were trapped inside the device.

**Table 1:** The composition of different types of leukocytes cells compared with whole blood volume and their nucleus to cytoplasmic (N/C) ratios^35–37^ to determine the nucleus size to justify their inability to get trapped in the device.

Cell type	Percent in blood	N/C ratio	Cell size (µm)	Nucleus size (µm)
Myeloblast	< 1	4:1	15–20	4.37
Promyelocyte	< 1	3:1	14–20	5.66
Myelocyte	< 1	2:1	12–18	7.5
Metamyelocyte	< 1	1.5:1	10–15	8.33
Neutrophils	60–70	3:1	10–15	4.16
Eosinophils	2–4	2:1	12–17	7.25
Basophils	0–1	3:1	10–14	4
Promonocyte	< 1	2–3:1	12–20	6.4
Monocyte	3–8	2–3:1	12–20	6.4
Lymphoblast	< 1	4:1	10–18	3.5
Lymphocyte	20–30	3–5:1	7–18	3.12
Plasma cell	< 1	1–2:1	8–20	9.33

## Conclusion and future directions

Microfluidics has been consistently evolving for different scientific applications and has enormous impacts on biomedical fields^3^. A novel and unique microfluidic isolation method using multifunctional microposts has been successfully demonstrated. The platform has demonstrated high capture efficiency at fast flow rates without clogging problems in experiments with microbeads and cancer cells-spiked human blood. In view of isolating relatively larger tumor cells from biological fluids, our strategy integrated microfluidic method with a distinct size-based cell capturing unit. While the overall flow rate for the sample flow through the device was high, maintaining a low velocity of fluid movement at the capture site was critical to retain the captured cell while keeping the shear stress on the cells at a bare minimum. These distinct features are critical in maintaining cell integrity and retrieving cells for further testing, an invaluable asset in the field of biomedical analyses for characterizing rare cells from biological fluids (liquid biopsies). The low number of cancer cells that we used for standardized testing mimics the clinical scenario in cancer patients who are typically carrying a low number of CTCs in their blood.

In future studies, investigations on viable cell releasing operations need to be performed, in addition to evaluating the impact of whole blood samples on device performance. Viable cell characterization can be utilized for advanced biological applications in mechanistic scientific studies, advanced systems biology, and precision medicine such as personalized drug testing. The present and novel microfluidic device principles have the potential for future diagnostic and therapeutic uses and molecular analysis of captured biological particles, however further rigorous, standardized human application studies are needed to accomplish a robust clinical implementation.

## Methods

### Microfluidic device fabrication

The microfluidic device was fabricated using standard soft-lithography techniques with SU8 master mold on a silicon substrate. The negative photoresist SU8 3025 (Micro Chem^®^, Bound Rock, TX) was used to prepare a silicon wafer to serve as the master mold for the polymeric filter device. Standard photolithography methods were followed. Considering different parameters (e.g., operation flow rates, typical cell sizes) the optimal microfluidic channel height was determined to be 30 µm ± 2 µm. Sylgard™ 184 elastomer polydimethylsiloxane (PDMS) (Dow Corning Inc. Midland, MI) and its curing agent were mixed in the required ratio and degassed to remove air pockets and then poured on the silicon master inside the Petri dish and cured at 75 °C for 3 h. Then the surface of the PDMS containing the microposts and a clean microscopic glass slide (1″ × 3″) were oxygen plasma-treated at 70 W for 50 s and then bonded while ensuring the absence of air pockets within the bonding layers. The bonded device was placed on the hot plate at 80 °C for 15 min to achieve stronger adhesion. The inlet and outlet ports were created using a 0.6 mm Tungsten Carbide Drill Bit on the PDMS, followed by bonding of PTFE tubes to the ports.

### Computational analysis of the flow profile in the microdevice

COMSOL Multiphysics^®^ version 5.3a finite element analysis software (COMSOL Inc., Burlington, MA) was used to computationally simulate the velocity profiles within the device, as demonstrated in Fig. 1c,d. The asymmetrical geometry of Design-I was achieved by bonding two partial circles with a radius of curvatures of 70 µm and 38 µm, respectively. Numerical analysis was performed to identify the optimal operating conditions and design for the microposts with wing-like geometry using COMSOL software (Fig. 2b,e). The operating conditions for the fluidic medium tested were like that of water, consisting of incompressible flow, inlet linear velocity of 12 mm/s, no-slip boundary conditions for all walls, and outlet pressure of zero.

### Platform design

The platform was designed using AutoCAD^®^ (Autodesk, Inc., San Rafael, CA) software comprising of 506 rows and 54 columns having a total of 27,324 post pairs with each pair having one trapping site. The chip comprises a height of 30 µm and a filter area of 45 mm × 15 mm. The geometry for Design I (Fig. 2a) was formed by fusing two partial circles of radius 37 µm and 90 µm (Supplementary information S2), respectively. Later this smooth curve was extended and contoured downwards to direct itself towards the consecutive trapping region to create Design II (Fig. 2d) with increased capture efficiency.

### Device characterization for flowrate optimization using microbeads and human cancer cells

Microfluidic platform was optimized for flowrate using FluoSpheres™ Polystyrene Microbeads (10 µm) (Cat#: F8833; Thermo Fisher Scientific) suspended in phosphate-buffered saline (PBS) (Cat#: 14190250; Thermo Fisher Scientific). For each testing, one hundred microbeads were picked individually by using a micropipette and spiked into 4 ml of PBS buffer. The microbead suspension was passed through the inlet port of the device. The microbeads captured (Fig. 2g) in the device were counted using bright-field microscopy. Capture efficiency (%) was calculated as a ratio of the number of microbeads caught in the device to the total number of microbeads in the input sample. Five repetitions of spiking experiments were performed for each flow rate. Testing was performed at nine different flow rates between 0.25 and 3.0 ml/min.

For biological device testing, human lung cancer cell lines A549 (ATCC^®^ CCL-185™, ATCC, Manassas, VA) were used. CTCs and cancer cell lines have shown to have a diameter over 10 µm^38,39^. This size difference can be exploited for size-based enrichment of CTCs from the blood as they are larger than the majority of other cells (e.g., red blood cells (RBCs), leukocytes)^38,39^. A549 human cancer cells were maintained in DMEM medium (Cat#: 11965118; Thermo Fisher Scientific) supplemented with 10% fetal bovine serum and 1% Penicillin–Streptomycin (Cat#: 15140122; Thermo Fisher Scientific). For device testing, cancer cells were grown to ~ 80% confluence. The cells were harvested using 0.05% Trypsin–EDTA (Cat#: 25300120; Thermo Fisher Scientific). For each testing, one hundred cancer cells were picked individually by using a micropipette and spiked into 4 ml of PBS buffer. Then, the cancer cell suspension was passed through the inlet port of the device. The cancer cells captured in the device were counted using bright-field microscopy. Capture efficiency (%) was calculated as a ratio of the number of cancer cells caught in the device to the total number of cancer cells in the input sample. Five repetitions of spiking experiments were performed for each flow rate. Testing was performed at nine different flow rates between 0.25 and 3.0 ml/min.

### Pre-clinical testing using human cancer cells spiked in human blood

The study protocol involving human blood samples was approved by the University of Missouri Institutional Review Board (MU IRB approval: #2010166). Written informed consent was obtained prior to blood collection and the blood samples from the human participants involved were obtained, transferred, and preserved for experiments in accordance with the declaration of Helsinki. Whole blood from healthy volunteers was drawn into K2EDTA vacutainer tubes. A specific number of human lung cancer cells (line A549) were spiked into the anti-coagulated whole blood. Spiked blood was passed through the inlet port of the device. The cancer cells captured in the device were counted using bright-field microscopy. To confirm that trapped cells are spiked cells, A549 cells were formalin-fixed, permeabilized, and stained with DAPI before spiking. A549 spiked and PBMCs spiked blood samples were independently processed with the device to determine differential capture efficacies in terms of cancer cells and WBCs.

### Isolation and labeling of PBMCs from healthy human blood

Whole blood (10 ml) from healthy donor was collected in K2EDTA tubes. Anti-coagulated blood was diluted (1:1) with PBS and carefully layered on top of Ficoll-Paque Plus reagent and subjected to density-gradient centrifugation at 2000 rpm for 20 min at room temperature with no brakes. The interphase of PBMCs between upper plasma phase and lower Ficoll phase was harvested, counted and final cell suspension of 1 × 10^6^ cells/ml was prepared in serum-free DMEM medium. For labeling, PBMCs were incubated with 2 µM CellTracker™ Green CMFDA dye (C2925; Thermo Fisher) at 37 °C for 30 min. Labelled PBMCs (1 × 10^6^ cells) and DAPI stained A549 cells (100 cells) were spiked into a fresh whole blood for device testing.

## Supplementary Information

Supplementary Information.
