# Supplementary material for: Clog-free high-throughput microfluidic cell isolation with multifunctional microposts
Source: Sci Rep. 2021 Aug 17;11:16685. doi: 10.1038/s41598-021-94123-6 (PMC8370995; doi:10.1038/s41598-021-94123-6)
Supplement: Supplementary file 1 — Supplementary Information. [file 41598_2021_94123_MOESM1_ESM.docx]

**Importance of secondary flow in the functionality of microfluidic device**

Secondary flow carries the fluid forward to subsequent capture sites. The intensity of secondary flow can be analysed using the Dean number (D_e_), a dimensionless parameter that defines secondary flow characteristics in a curved channel, as follows:

$$D_{e}=R_{e}. \sqrt{\frac{D_{h}}{R}}$$

The Dean number (D_e_) is a function of the Reynolds number (R_e_), the hydraulic diameter (D_h_) for a rectangular channel, and the radius of curvature (R)^26, 28, 29^. The Dean number (D_e_) is directly proportional to the Reynolds number (R_e_) and inversely proportional to the square root of the radius of the curvature (R). A change in these parameters alters the intensity of secondary flow in the microfluidic channel. The Reynolds number for a rectangular channel is given as follows:

$$R_{e}=\frac{L.V_{avg}.\rho}{\mu}$$

The Reynolds number is proportional to characteristic length (L), fluid average velocity (V_avg_), Fluid density (ρ) and inversely proportional to Viscosity of the fluid used (µ). The fluid used here is either saline solution or diluted blood. To keep the Reynolds number in the range to produce laminar flow inside the device the blood dilution ratio is 1:5 (Blood:PBS). The hydraulic diameter (D_h_) for a rectangular channel is as follows:

$$D_{h}=\frac{2wh}{w+h}$$

The width (w) and height (h) of the rectangular channel will have a significant impact on the hydraulic diameter (D_h_). By modifying these parameters, we designed a micropost with a sufficiently large Dean number (D_e_) to achieve a high secondary flow to aid the particle movement around the post. **Main Fig.1b** shows the microscopic image of the micropost arrangement within the device with the capture site that has a 7 µm gap, and an additional 14 µm by-passing channel. This distinct design results in an asymmetrical flow pattern. The movement of particles around these high-aspect ratios (h/w) of the microfluidic channel can be explained by the rotation-induced lift force (F_Ω_) which is a resulting factor of secondary flow^30-32^. Firstly, the particles experience shear-induced lift force^30, 31^ (F_S_) and move towards the sidewalls of the microposts. Then due to rotation-induced lift force (F_R_), the particles are pushed away from the walls and an equilibrium is achieved along the long-curved surfaces of the micropost. Due to our unique micropost design the rotation-induced lift force (F_R_) is predominant, with larger particles experiencing more force due to their density that directs them away from the walls of the micropost towards the capture site^29, 33^ **(Supplementary Fig. S1.b)**. For a particle to be considered sufficiently large to experience this force, it must have a diameter greater than 72% of the channel depth^31^. In addition, any smaller particles with diameters less than 27% of the channel depth will not experience enough force to be directed towards the capture site^31^.

The asymmetrical geometry of Design-I was achieved by bonding two partial circles with radius of curvatures of 7$0$µm and 38 µm, respectively. The rationale behind this design was to create an unbalanced flow pattern on both sides of the microposts by using a standard circular and equilateral design^14^. Numerical analysis was performed for Design-I to identify the optimal operating conditions and design for the microposts with wing-like geometry using COMSOL software **(Main Fig. 2b)**. The operating conditions for the fluidic medium used were similar to that of water, consisting of incompressible flow, inlet linear velocity of 12 mm/sec, no-slip boundary condition for all walls, and an outlet pressure of zero. The representation of the velocity profile in the separation zone of the two by-passing channels as shown in the thermal graph in **Main Fig. 2b**. High velocity was observed above the capture site, serving the purpose to guide the laterally displaced particle towards the capture site **(Main Fig. 2c)**. To further improve the capture efficiency, the hydrodynamic profiles were then enhanced in the next-generation Design-II.

The computational simulations then assisted to further improve our device from a pilot Design-I to next-generation Design-II **(Main Fig.2e)**. Design-II is the augmented version of Design-I to improve capture efficiency by increasing the angle of the curvature of the micropost. The geometry was formed by fusing two partial circles of radius 37 µm and 90 µm, respectively. This modification alters the by-passing channel and appears more guiding towards the capture site. In comparison to Design-I, this leads to higher fluidic velocity above the capture site **(Main Fig. 2e)**. Once a particle is trapped at the capture site **(Main Fig. 2f)**, inertial (drag) force increases (Simulation analysis in the last section of the supplementary section) at the higher curvature angle of the micropost. This inertial force ensures the movement of the remaining particles through the by-passing channel towards the next level of capturing units. Consequently, this alignment of several subsequent capturing levels results in a higher probability of particle isolation. The larger particles experience rotation-induced lift force (F_Ω_) for longer distance when compared to Design I while passing through the 14 µm sized by-passing channel and again at the separation zone. In summary, in our unique microfluidic devices the rotation-induced lift force (F_Ω_) was used to separate large particles (such as tumor cells) from the main streamline and isolate them at the distinct capture site with great efficiency applying advanced fluid dynamics.

**Comparison of two micropost array designs.**

**Supplementary Fig. S2** shows the two designs of micropost array and their sectional dimensions of selective regions of the micropost. There are two main reasons for the efficiency of Design-II to be better. One of the reasons is the length of trapping channel. In comparison, the trapping channel length of Design-II **(Supplementary Fig. S2.d)** is greater than Design-I **(Supplementary Fig. S2.b)** and this results in the lesser probability that a larger trapped particle would squeeze through the trapping zone. The other reason involves the radius of curvature near the trapping zone. From **Supplementary Fig. S2.b and S2.d** the radius of curvature of Design-I is larger than Design-II thus the effect of drag force is higher on an unsettled particle in trapping zone forcing it through the bypass channel. This can be verified from **Main Fig. 3** showing that the Design-II efficiency is improved due to inclusions of these geometrical parameters.

**Change in velocity profile around a trapped particle.**

**Supplementary Fig. S3** shows the computational analysis of the velocity profile of Design-I. When a particle is trapped as stated in the “Importance of secondary flow in the functionality of microfluidic device” section that the drag forces increase in the bypass zone when a particle is trapped facilitating for it to rest and remain undisturbed can be verified in this simulation. The velocity indicated in the red box region (near trapped particle) is slightly larger than that of the black box region (near no trapped particle) in **Supplementary Fig. S3**, thus verifying that once the particle is trapped the remaining incoming particles are diverted towards the slightly stronger bypass region and not disturb an already trapped particle.

**Supplementary Figures**


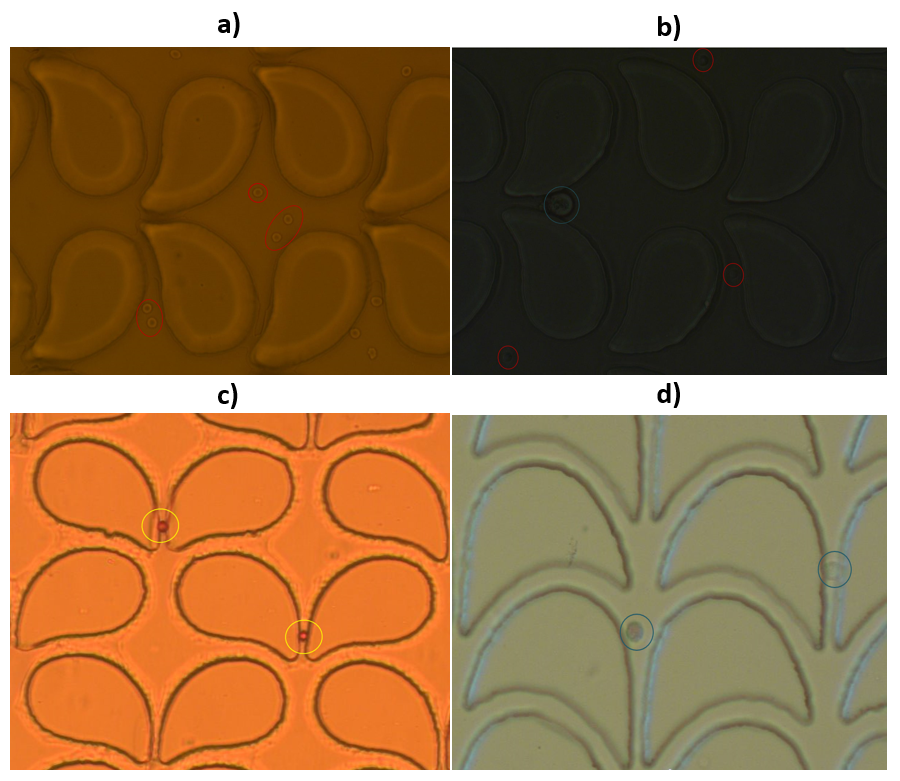


**Fig. S1 Particle size comparison in the device. (a)** Microscopic brightfield image of RBCs scattered in the device micropost array (Design-I) (indicated by red circles). **(b)** Comparison of cell sizes between a cancer cell (blue circle) and RBCs (red circles) in the device. The RBCs stay when the device is not flushed with enough PBS saline solution. **(c)** Microscopic brightfield image of trapped microbeads in device micropost array (Design-I) (indicated by yellow circles). **(d)** Microscopic brightfield image of cancer cells in the device micropost array (Design-II) (indicated by blue circles) showing a longer trapping channel in comparison to Design-I.


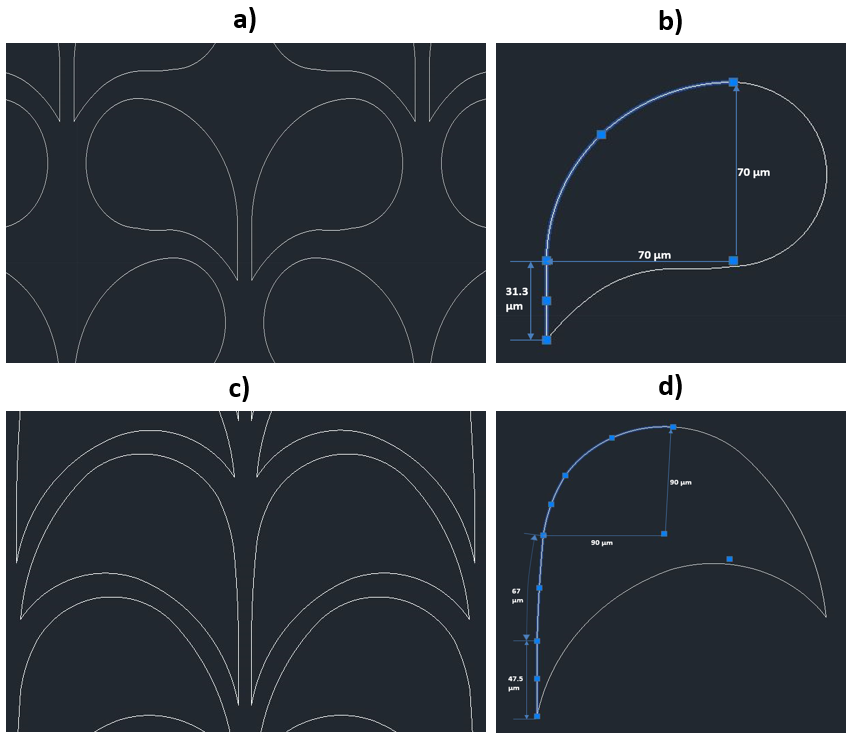


**Fig. S2 Micropost size comparison of the two designs used. (a)** AutoCAD drawing image of Design-I micropsot array. **(b)** Sectional view of one micropost array of Design-I showing the dimensions of trapping channel and radius of the curvature of trapping zone. **(c)** AutoCAD drawing image of Design-II micropsot array. **(d)** Sectional view of one micropost array of Design-II showing the dimensions of trapping channel and radius of the curvature of trapping zone.


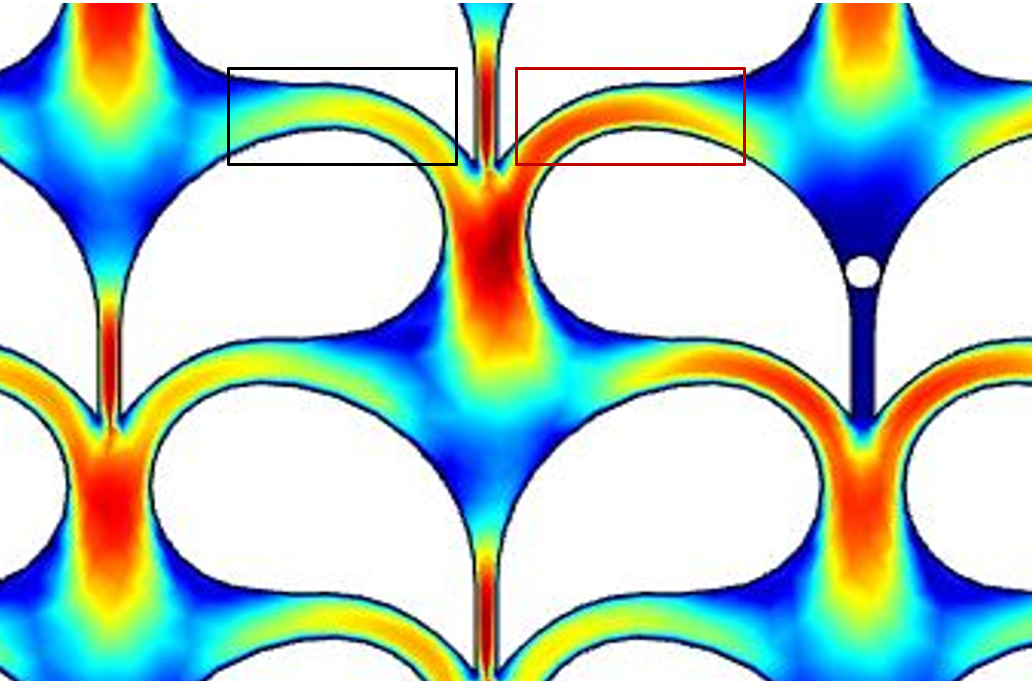


**Fig. S3 Changes in bypass velocity around a trapped particle.** Computational analysis of velocity profile at bypass path near a trapped particle (Red box) and velocity profile at bypass path near a empty trapping zone (Black box).
